# Roles of Post-translational Modifications in Spinocerebellar Ataxias

**DOI:** 10.3389/fncel.2018.00290

**Published:** 2018-09-19

**Authors:** Linlin Wan, Keqin Xu, Zhao Chen, Beisha Tang, Hong Jiang

**Affiliations:** ^1^Department of Neurology, Xiangya Hospital, Central South University, Changsha, China; ^2^National Clinical Research Center for Geriatric Diseases, Central South University, Changsha, China; ^3^Key Laboratory of Hunan Province in Neurodegenerative Disorders, Central South University, Changsha, China; ^4^Laboratory of Medical Genetics, Central South University, Changsha, China; ^5^Parkinson’s Disease Center of Beijing Institute for Brain Disorders, Beijing, China; ^6^Collaborative Innovation Center for Brain Science, Shanghai, China; ^7^Collaborative Innovation Center for Genetics and Development, Shanghai, China; ^8^Department of Neurology, Xinjiang Medical University, Ürümqi, China

**Keywords:** post-translational modification, spinocerebellar ataxias, protein, pathogenesis, therapy

## Abstract

Post-translational modifications (PTMs), including phosphorylation, acetylation, ubiquitination, SUMOylation, etc., of proteins can modulate protein properties such as intracellular distribution, activity, stability, aggregation, and interactions. Therefore, PTMs are vital regulatory mechanisms for multiple cellular processes. Spinocerebellar ataxias (SCAs) are hereditary, heterogeneous, neurodegenerative diseases for which the primary manifestation involves ataxia. Because the pathogenesis of most SCAs is correlated with mutant proteins directly or indirectly, the PTMs of disease-related proteins might functionally affect SCA development and represent potential therapeutic interventions. Here, we review multiple PTMs related to disease-causing proteins in SCAs pathogenesis and their effects. Furthermore, we discuss these PTMs as potential targets for treating SCAs and describe translational therapies targeting PTMs that have been published.

## Introduction

Post-translational modifications (PTMs) of proteins are crucial regulators of protein properties which can generate various types of proteins with different functions (Ratovitski et al., 2017). These modifications mostly arise from covalently attaching functional groups to specific amino acid residues including phosphate, acetate, and other chemical moieties, while another modification called proteolytic cleavage results from removing or separating specific portions of proteins (Karve and Cheema, 2011; Matos et al., 2017) (**Table 1**). PTMs, such as phosphorylation, acetylation, ubiquitination, SUMOylation, and proteolytic cleavage, can modulate the turnover, localisation, activity, and interaction of proteins (Ju et al., 2014). Thus, PTMs play pivotal roles in regulating multiple cellular pathways to further modulate pathogenesis in a number of diseases. For example, PTMs have been shown to modulate pathogenesis in cardiovascular diseases, neurological diseases, diabetes, liver cirrhosis, and cancer (Mendez et al., 2010; Modol et al., 2011; Orr, 2012; Xie et al., 2017; Zhang et al., 2017). Studying the roles of PTMs in disease processes can facilitate a better understanding of pathogenesis and uncover therapeutic targets for these diseases.

**Table 1:** Summary of common PTMs, their functional groups and possible amino acid sites (Klemm, 1984; Violante et al., 2001; Karve and Cheema, 2011; Hickey et al., 2012).

PTMs and functional group	Amino acid									
	
	Alanine	Arginine	Asparagine	Aspartic acid	Cysteine	Glutamine	Glutamic acid	Glycine	Histidine	Isoleucine
Acetylation (Acetyl)	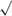	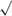		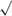			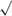	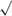	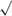	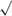
Carbonylation (Carbonyl)		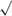		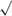	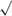		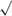		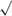	
Glycosylation (Glycan)		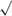	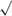							
Hydroxylation (Hydroxyl)			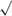	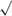						
Methylation (Methyl)		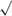				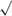			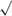	
Nitration (Nitryl)										
Palmitoylation (Palmitate)					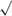					
Phosphorylation (Phosphate)										
Sulphation (Sulphate)										
Proteolytic cleavage	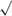	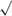	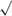	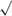	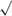	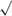	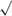	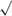	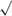	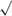
SUMOylation (SUMO)										
Transglutamination (Acyl)										
Ubiquitination (Ubiquitin)										

**PTMs and functional group**	**Amino acid**									
	
	**Leucine**	**Lysine**	**Methionine**	**Phenylalanine**	**Proline**	**Serine**	**Threonine**	**Tryptophan**	**Tyrosine**	**Valine**

Acetylation (Acetyl)	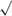	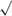			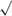	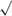	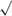			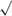
Carbonylation (Carbonyl)		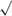			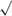		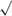			
Glycosylation (Glycan)						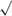	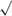	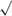	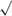	
Hydroxylation (Hydroxyl)		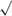		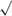	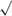			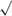	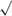	
Methylation (Methyl)		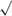	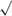			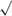				
Nitration (Nitryl)								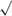	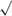	
Palmitoylation (Palmitate)										
Phosphorylation (Phosphate)						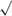	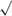		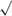	
Sulphation (Sulphate)									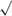	
Proteolytic cleavage	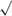	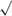	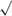	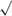	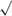	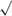	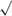	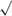	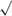	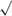
SUMOylation (SUMO)		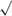								
Transglutamination (Acyl)		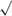								
Ubiquitination (Ubiquitin)		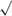								


Spinocerebellar ataxias (SCAs) are a group of heterogeneously neurodegenerative diseases characterised by the progressive disequilibrium of motor coordination, cerebellar atrophy and other various non-ataxia manifestations (Rossi et al., 2014). To date, more than 40 subtypes of SCAs have been reported^1^, including dentatorubral-pallidoluysian atrophy (DRPLA), which is classified as a specific subtype (Durr, 2010). The most common SCAs are caused by (CAG)_n_ expansions in a specific gene that encode expanded polyglutamine (polyQ) tracts. Some SCAs arise from a non-coding repeat expansion, and others result from point mutations or insertions/deletions in their respective gene (Pandolfo and Manto, 2013). The pathogenesis of SCAs is most commonly correlated with aggregation of mutant proteins containing polyQ, such as SCA1, SCA2, SCA3, SCA6, SCA7, SCA17, and DRPLA. In addition, some specific proteins are also implicated in non-polyQ SCAs, such as mutant KCNC3 in SCA13 (Gallego-Iradi et al., 2014). Recent studies have revealed that the PTMs of proteins correlated with neurodegenerative diseases modulate their properties, including intracellular distribution, activity, stability, aggregation, protein interaction network and clearance (Sambataro and Pennuto, 2017). Therefore, investigation into the relationship between SCAs and PTMs may shed light on SCA pathogenesis. For instance, one characteristic feature of polyQ diseases is selective neurodegeneration, yet the mechanism remains unclear. Two possible factors contributing to selective neuronal impairment are the abnormal subcellular localisation of polyQ proteins and the change in their folding and function (Sambataro and Pennuto, 2012). PTMs are shown to regulate protein properties including their intracellular localisation and functions (Sambataro and Pennuto, 2017). Therefore, understanding the PTMs in polyQ SCAs may yield important clues into mechanisms behind the selective neuronal damage. What’s more, these PTMs involved in SCA disease processes can help us discover novel therapeutic targets. Till now, many studies have investigated this topic.

Here, we review PTMs that have been proven to serve regulative roles in SCA pathogenesis; these PTMs include phosphorylation, ubiquitination, SUMOylation, proteolytic cleavage, transglutamination, acetylation and *N*-glycosylation. We describe how these PTMs might modulate protein characteristics and further affect the disease process (**Table 2**). The potential and limitation of these PTMs as therapeutic targets for SCAs are also discussed.

**Table 2:** Summary of post-translational modifications (PTMs) in proteins related to SCAs.

Disease	Protein	PTM	Reacting site	Interactor	Mechanism	Effect on disease process
SCA1	ATXN1	Phosphorylation	S776	PKA	Decrease the degradation of ATXN1;	Stimulative
				MSK1	Increase the stability and aggregation of ATXN1.	
				PP2A		
			S239	NLK	nd^a^	Stimulative
		Ubiquitination	K589	UbcH6	Increase the degradation of ATXN1;	Suppressive
			nd	CHIP	Increase the degradation of ATXN1.	Suppressive
				MDM2		
				A1Up		
				FAT10		
				RNF4		
		SUMOylation	K16	SUMO-1	Increase the aggregation of ATXN1.	Stimulative
			K194			
			K610			
			K697			
			K746			
		Transglutamination	nd	TG2	Increase ATXN1 aggregate formation and Cab recruitment to ATXN1.	Stimulative
SCA2	ATXN2	Phosphorylation	nd	Cdk5–p25	Increase the degradation of ATXN2.	Suppressive
SCA3	ATXN3	Phosphorylation	S12	nd	Decrease aggregation and cytotoxicity of ATXN3.	Suppressive
			S29	CK2	Increase the nuclear uptake of ATXN3.	Stimulative
				GSK3β		
			S55	nd	Modulate the catalytic activity of ATXN3	nd
			T60			
			S236	CK2	Increase inclusion formation, nuclear localisation and stability of ATXN3.	Stimulative
			S260			
			S261			
			S340			
			S352			
			S256	CK2	Increase inclusion formation, nuclear localisation and stability of ATXN3.	Stimulative
				GSK 3β	Decrease the aggregation of ATXN3.	Suppressive
SCA3	ATXN3	Ubiquitination	K8	nd	May modulate the catalytic activity of ATXN3.	nd
			K117	nd	Activate the ATXN3 function as DUB and increase the degradation of ATXN3.	Suppressive
			nd	Parkin	Increase the degradation of ATXN3.	Suppressive
				CHIP		
				E4B		
		SUMOylation	K166	SUMO-1	Increase the stability of ATXN3 and cell apoptosis.	Stimulative
			K356	SUMO-1 SUMO-2	Increase the degradation and decrease the aggregation of ATXN3.	Suppressive
		Proteolytic cleavage	D241	Caspase	Increase the formation of ATXN3 fragments and aggregates.	Stimulative
			D244			
			D248			
			D208	Calpain	Increase the formation of ATXN3 fragments and aggregates.	Stimulative
			S256			
SCA7	ATXN7	SUMOylation	K257	nd	Decrease the aggregation of ATXN7 and the cell apoptosis.	Suppressive
		Proteolytic cleavage	D266	Caspase	Increase the formation of ATXN7 fragments and aggregates.	Stimulative
			D344			
		Acetylation	K257	HDAC3	Increase the aggregation of ATXN7 fragments.	Stimulative
SCA13	KCNC3^R420H^ proteins	*N*-glycosylation	NKT	nd	Important for the expression of K+ currents and trafficking of KCNC3^R420H^ proteins to the plasma membrane.	Suppressive
			NIT			
SCA14	MARCKS	Phosphorylation	S159	γPKC	Important for regulation of the membrane trafficking, actin cytoskeleton and macropinocytosis.	Suppressive
			S163			
			S167			
			S170			
DRPLA	ATN1	Phosphorylation	S734	JNK	Involved in insulin/IGF-I signal pathway and maintain neuronal viability.	Suppressive
		Ubiquitination	nd	nd	nd	Stimulative
		SUMOylation	nd	SUMO-1	Increase the aggregation of ATN1.	Stimulative
		Proteolytic cleavage	D109	Caspase	Increase the formation of ATN1 fragments and regulate the intracellular localisation of the fragments or other roles (need further studies)	nd
PolyQ	Histone	Acetylation	H3:	HATs	Modulate gene transcription.	Suppressive
			K9	HDACs		
SCAs			K14	CBP		
			K18	P300		
			K23	P/CAF		
			H4:			
			K5			
			K8			
			K12			
			K16			


*^a^nd, not defined.*

## Phosphorylation

Phosphorylation is a vital PTM in which a phosphate group is added to an amino acid. This PTM can regulate protein–protein interactions, cellular metabolism, protein degradation, and enzyme reactions (Karve and Cheema, 2011). If the activity and function of the disease-causing protein are controlled by phosphorylation, phosphorylation might play a significant role in the disease.

**FIGURE 1:**
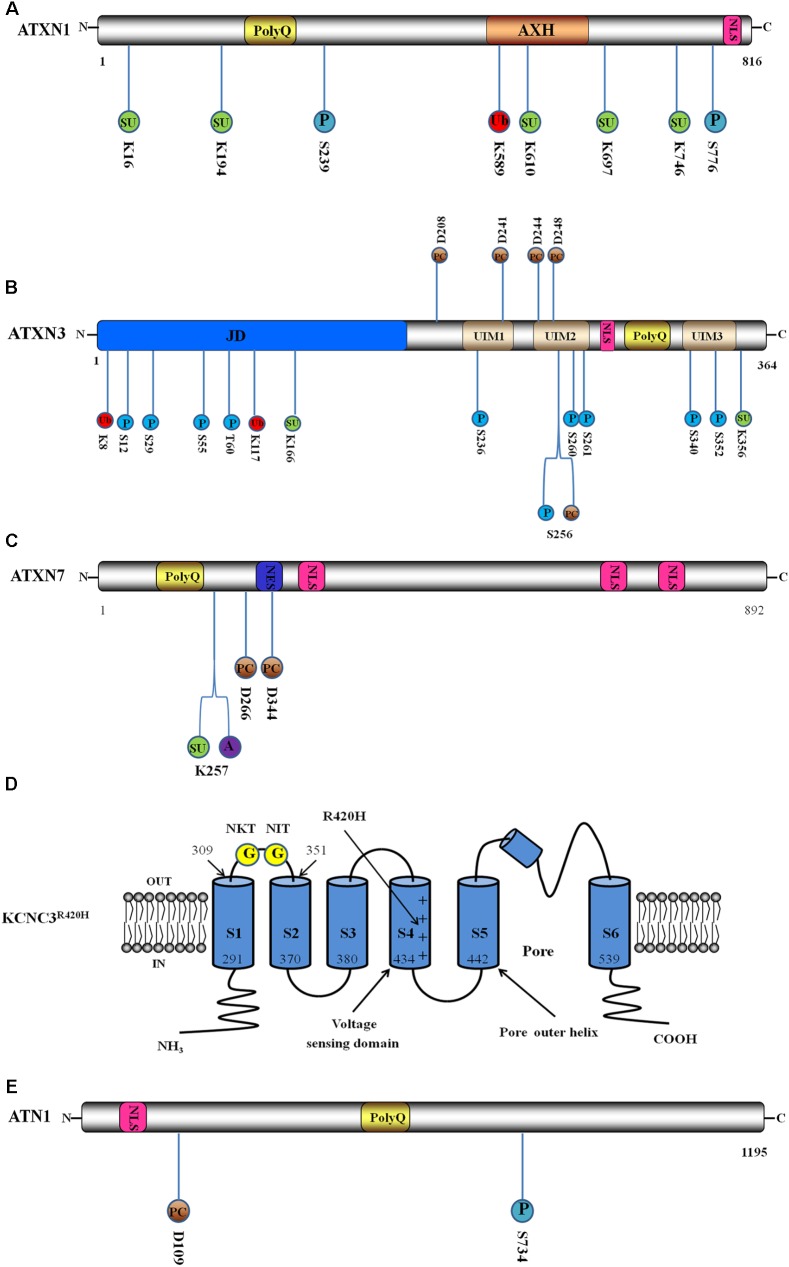
Schematic interpretation of direct pathogenesis-related mutant protein constructs and post-translational modification (PTM) sites. Blue circle stands for phosphorylation site, red circle stands for ubiquitination site, green circle stands for SUMOylation site, brown circle represents proteolytic cleavage site, purple circle represents acetylation site, and yellow circle represents *N*-glycosylation site. The number below or above the circle represents the position of PTM sites. **(A)** Domain architecture and PTM sites of ATXN1: ATXN1 incorporates the expanded polyQ stretch, the globular ataxin1/HBP1 domain (AXH) and a nuclear localisation sequence (NLS). Here are illustrated two phosphorylation sites, a ubiquitination site and five SUMOylation sites. **(B)** Domain structure and PTM sites of ATXN3: ATXN3 incorporates the globular Josephin domain (JD), a flexible C-terminal tail containing three ubiquitin interaction motifs (UIMs), an NLS and the expanded polyQ. Here are illustrated ten phosphorylation sites, two ubiquitination sites, two SUMOylation sites and five proteolytic cleavage sites. **(C)** Domain structure and PTM sites of ATXN7: ATXN7 incorporates the expanded polyQ, a nuclear export signal (NES) and three NLS. Here are shown a SUMOylation site, an acetylation site and two proteolytic cleavage sites. **(D)** Schematic interpretation of the Kv3.3 channel in R420H variant (KCNC3^R420H^) and PTM sites. Here are illustrated the six transmembrane segments and pore re-entrant loop. The numbers show the positions of the ending and beginning amino acids of S1 and S2 domains. Location of R420H-mutation is indicated by the long arrow. The two *N*-glycosylation sites are located in the S1–S2 extracytoplasmic loop. Adapted from Waters et al. (2006). **(E)** Domain structure and PTM sites of ATN1. ATN1 incorporates an NLS and the expanded polyQ. Here are shown a phosphorylation site and a proteolytic cleavage site.

SCA1 is caused by a (CAG)_n_ expansion in the ataxin1 (ATXN1)-encoding gene, which results in a polyQ expansion in ATXN1. ATXN1 is a DNA-binding protein incorporating the expanded polyQ stretch, the globular ataxin1/HBP1 domain (AXH) and a nuclear localisation sequence (NLS) (**Figure 1A**). In addition, wild-type ATXN1 contains from 6 to 44 polyQ repeats, while the repeat number is from 39 to 83 in mutant ATXN1 (Zoghbi and Orr, 2009). At least seven phosphorylation sites have been identified *in vivo* in ATXN1 (Huttlin et al., 2010), and phosphorylation at serine residues 776 (S776) and 239 (S239) is believed to significantly affect SCA1 pathogenesis (**Figure 1A**) (Ju et al., 2013; Park et al., 2013). Phosphorylation at S776 of polyQ-expanded ATXN1 might stabilise ATXN1 by blocking its proteolysis (Orr, 2012). In addition, it also regulates the interaction between ATXN1 and other cellular proteins. S776 phosphorylation promotes binding to the cellular signalling protein family 14-3-3, which prevents pS776 dephosphorylation and protects ATXN1 from proteolysis. In addition, S776 phosphorylation inhibits ATXN1 localisation to the nucleus by masking the nuclear localisation signal, which also prevents dephosphorylation (Lai et al., 2011). S776 phosphorylation is also essential for the formation of the ATXN1-CIC complex, which is vital for ATXN1 maintenance and stabilisation; thus, this complex might be relevant to SCA1 disease progression (Ju et al., 2014). Binding to U2AF65, a constitutive component of the spliceosome, is impeded but not eliminated by the phosphorylation of S776, and this reduces ATXN1 binding to the large spliceosome machinery, leaving ATXN1 conformationally extended and vulnerable to self-association and aggregation (Menon et al., 2012). In addition, the phosphorylation status of S776 is critical to the interaction between ATXN1 and RBM17 (splicing factor RNA-binding motif protein 17), an interaction that contributes to the neuropathology of SCA1 (Lim et al., 2008). For the regulation of phosphorylation, cyclic AMP-dependent protein kinase (PKA) and mitogen- and stress-activated protein kinase-1 (MSK1) phosphorylate ATXN1 at S776 (Jorgensen et al., 2009; Park et al., 2013). In addition, the RAS-MAPK-MSK1 signalling pathway, which includes the components ERK1, ERK2, MEK2, MEK3, and MEK6, serves as an important regulator of S776 phosphorylation and ATXN1 stability (Park et al., 2013). Conversely, protein phosphatase 2A (PP2A) can dephosphorylate ATXN1-pS776, playing an important role in regulating S776 phosphorylation (Lai et al., 2011). One study suggested that ATXN1 could be phosphorylated at S239 by Nemo-like kinase (NLK), an ATXN1 interactor that modulates the toxicity of SCA1; this hypothesis is supported by NLK overexpression in a *Drosophila* model, which increased ATXN1 disease-related phenotypes (Ju et al., 2013). However, the specific role of S239 phosphorylation in the pathogenesis of SCA1 requires further studies.

SCA2, one of the nine polyQ neurodegenerative diseases, is caused by aberrant expansion of the CAG repeat in the ATXN2-encoding gene. ATXN2 is a member of the Like-Sm (LSm) protein family and contains the expanded polyQ stretch, the LSm domain, the LSm-associated domain, proline-rich domains and the PAM2 domain. ATXN2 contains from 13 to 31 polyQ stretches in healthy individuals, but SCA2 patients possess more than 31 repetitions (Magana et al., 2013). SCA2 pathogenesis is linked to ATXN2 phosphorylation by cyclin-dependent kinase 5-p25 (Cdk5–p25), which induces ATXN2 degradation (Asada et al., 2014). Thus, Cdk5–p25 may play a protective role in SCA2 pathogenesis.

SCA3 (also called Machado-Joseph Disease, MJD), is caused by CAG repeat expansion in the ATXN3-encoding gene. ATXN3 is a deubiquitinating enzyme (DUB) consisting of the globular Josephin domain (JD), a flexible C-terminal tail containing three ubiquitin interaction motifs (UIMs), an NLS and the expanded polyQ stretch. The range of polyQ is from 12 to 44 in healthy individuals but from approximately 55 to 80 in MJD patients (Li et al., 2015) (**Figure 1B**). Several phosphorylation sites have been identified (S12, S29, S55, T60, S236, S256, S260, S261, S340, and S352) in ATXN3: S12, S29, S55, and T60 are located in the catalytic JD (aa 8–168), which is the N-terminal domain of ATXN3; S236 is located in the first UIM; S256, S260, and S261 are located in the second UIM; and S340 and S352 are located in the third UIM (**Figure 1B**) (Fei et al., 2007; Tao et al., 2008; Mueller et al., 2009; Pastori et al., 2010; Matos et al., 2016; Kristensen et al., 2017). A recent study showed that phosphorylation at S12 decreased the DUB activity, aggregation and cytotoxicity of ATXN3 (Matos et al., 2016) and that this site might be a novel therapeutic target for SCA3. Protein casein kinase 2 (CK2) and glycogen synthase kinase 3β (GSK3β) phosphorylate ATXN3 at S29, which promotes ATXN3 nuclear localisation and thus contributes to the pathogenesis of SCA3 (Pastori et al., 2010). Phosphorylation of the remaining sites in the JD, S55, and T60, may modulate catalytic activity. Increased phosphorylation of S55 has been detected in polyQ-expanded ATXN3, which may contribute to disease processes; however, more studies are needed to determine a specific effect. In addition to S29, CK2 can also phosphorylate sites S236, 256, 260, 261, 340, and 352 in ATXN3, altering the inclusion formation, nuclear localisation and stability of ATXN3 (Tao et al., 2008; Mueller et al., 2009), which indicates that CK2-dependent phosphorylation stimulates SCA3 pathogenesis. However, phosphorylation at S256 by GSK 3β inhibits ATXN3 aggregation, which plays a protective role in SCA3 pathophysiology (Fei et al., 2007). We conclude that phosphorylation of the same sites by different kinases produces different effects on protein activities.

**FIGURE 2:**
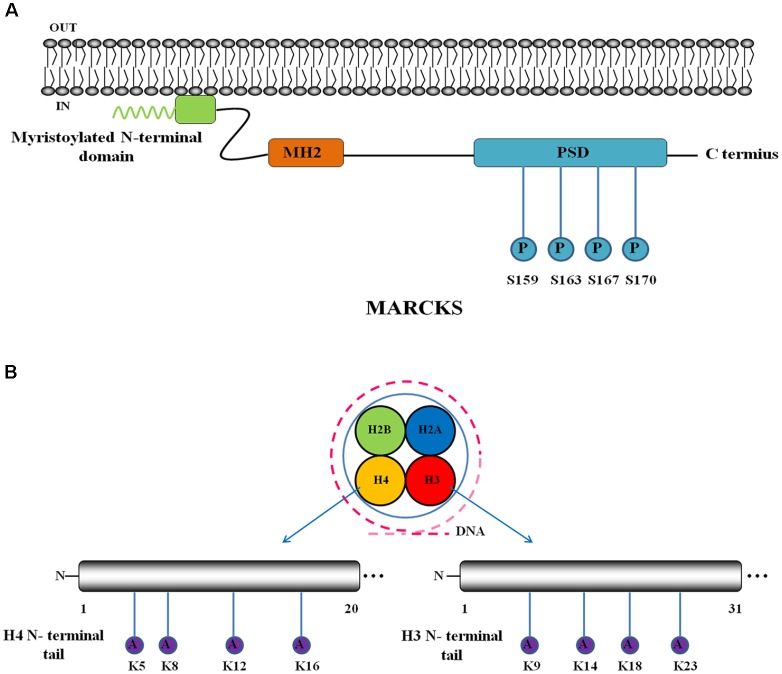
Schematic interpretation of non-direct pathogenesis-related protein constructs and post-translational modification (PTM) sites. Blue circle stands for phosphorylation site and purple circle represents acetylation. **(A)** Domain structure and PTM sites of MARCKS: MARCKS incorporates a myristoylated N-terminal domain, an MH2 domain and a phosphorylation site domain (PSD) with four phosphorylation sites. Adapted from Fong et al. (2017). **(B)** Schematic interpretation of PTM sites in histone N-terminal tails: H3 and H4 have four acetylation sites in their N-terminal tails respectively.

SCA14 is reported to arise from missense mutations in the gene encoding protein kinase Cγ (γPKC) (Chen et al., 2005), which can be activated by TPA (12-*O*-tetradecanoylphorbol 13-acetate) and regulates membrane trafficking, the actin cytoskeleton and macropinocytosis. A mutant form of γPKC that is linked to SCA14 cannot perform these regulatory activities because of reduced phosphorylation of its plasma membrane substrate MARCKS (myristoylated alanine-rich C-kinase substrate) (Yamamoto et al., 2014). MARCKS (**Figure 2A**) contains three conserved regions: a myristoylated N-terminal domain, an MH2 domain and a phosphorylation site domain (PSD) with four phosphorylation sites (S159, S163, S167, and S170) (Fong et al., 2017). Aberrant phosphorylation of MARCKS may be involved in the pathogenesis of SCA14, and further studies are required.

Dentatorubral-pallidoluysian atrophy is specific subtype of SCA caused by an expansion of the CAG trinucleotide repeat in the atrophin-1 (ATN1) gene; the number of repeats ranges from 3 to 36 in healthy individuals to 49 to 88 copies in DRPLA patients (Schols et al., 2004; Durr, 2010). ATN1 incorporates an NLS and the expanded polyQ tract (Okamura-Oho et al., 1999) (**Figure 1E**). *In vitro* experiments provided evidence of ATN1 phosphorylation, a process primarily mediated by c-Jun NH2-terminal kinase (JNK) at S734 (**Figure 1E**). However, expanded polyQ might inhibit the affinity of JNK for ATN1 and cause that ATN1 is slowly phosphorylated in DRPLA patients. Aberrant phosphorylation of ATN1 might delay the insulin/IGF-I signalling pathway, a pathway which ATN1 is involved in and is vital to maintaining neuronal viability (Okamura-Oho et al., 1999; Okamura-Oho et al., 2003).

In conclusion, phosphorylation can alter the stability, degradation, aggregation or nuclear uptake of mutant proteins and thus may be protective or toxic for SCA pathogenesis. In addition, the phosphorylation status of some specific proteins involved in SCAs pathogenesis is critical to pivotal pathways; for example, phosphorylation of MARCKS is essential for membrane trafficking, actin cytoskeleton regulation and micropinocytosis. Based on such roles, some exploratory translational therapies related to phosphorylation in SCAs have been examined. Park et al. (2013) found that downregulating some components of the RAS-MAPK-MSK1 signalling pathway, such as RAS, ERK1, ERK2, and MAPK, suppresses ATXN1 levels and neurotoxicity in *Drosophila* and mouse models. Hearst et al. (2014) designed a therapeutic polypeptide, Synb1-ELP-PKI, which contains a thermally responsive elastin-like peptide (ELP) carrier, a PKA inhibitory peptide (PKI) and a cell-penetrating peptide (Synb1), to inhibit PKA based on phosphorylation at S776 by PKA. They found that treatment with Synb1-ELP-PKI decreased phosphorylation on S776 and suppressed the formation of intranuclear inclusions containing mutant ATXN1 in cell culture. In addition, this peptide was verified to improve SCA1 Purkinje cell morphology in cerebellar slice cultures, and the validity of intranasal delivery of the peptide to mouse brains has been demonstrated. However, most of these explorations are still in primary *in vitro*-level or model-level stages, and clinical drug trials are required before these therapeutic explorations become reliable. In addition, how to ensure accurate regulation on a specific residue is still a challenge due to the diverse effects of phosphorylation on different residues.

## Ubiquitination

Ubiquitination is a reversible PTM that occurs by forming an isopeptide bond between the K residues of a target protein and the C-terminal glycine of ubiquitin (Faggiano et al., 2015) and is regulated by numerous related enzymes, including the ubiquitin-activating enzyme (E1), the ubiquitin-conjugating enzyme (E2), the ubiquitin ligase (E3), and DUBs. An additional ubiquitylation enzyme has been recently identified (Koegl et al., 1999): E4, a novel conjugation factor known as yeast Ufd2, is required for polyubiquitin chain assembly in conjunction with E1, E2, and E3. Ubiquitination labels target proteins for degradation primarily by the ubiquitin proteasome system (UPS) (Hershko and Ciechanover, 1998) and autophagy, which have important regulatory functions in protein homeostasis. Because impaired protein degradation is always involved in SCA pathogenesis, ubiquitination is also significant in SCA pathogenesis.

A proteasome inhibition study (Cummings et al., 1999) confirmed the role of the UPS in ATXN1 degradation; inhibiting proteasome increases ATXN1 aggregation, and mutant ATXN1 is more resistant to ubiquitin-mediated degradation. UbcH6, a class III ubiquitin-conjugating enzyme (E2), ubiquitinates polyQ-mutated ATXN1 and enhances its subsequent degradation, which can uniquely be accomplished in the absence of E3 ligases (Hong et al., 2008), whereas most E3 ligases are indispensable for the ubiquitination function of the E2 conjugating enzyme. UbcH6 interacts with ATXN1 at K589 (**Figure 1A**) in the AXH domain of ATXN1, and a lysine 589 to arginine substitution impairs degradation, thus enhancing ATXN1 aggregation (Kang et al., 2015). Furthermore, the function of ATXN1 as a transcriptional repressor was suppressed by UbcH6 by modulating ATXN1 degradation (Lee et al., 2008). Because the repression activity of ATXN1 has a vital role in SCA1 pathogenesis, the effect of UbcH6 on transcriptional repression might be a potential therapeutic target. CHIP (C-terminal of Hsc70 interacting protein), which is also known as the STIP1 homology and U-box containing protein 1 (STUB1) (Shi et al., 2013), acts as a molecular co-chaperone through the TPR domain and as an E3 ubiquitin ligase through the U-box domain, linking the cellular folding-refolding machinery with the UPS (McDonough and Patterson, 2003). CHIP interacts with the molecular chaperone Hsp70, promoting Hsp70 induction and removing excess Hsp70 (Qian et al., 2006). This interaction enhances the ubiquitylation and degradation of misfolded proteins through the UPS (McDonough and Patterson, 2003). CHIP directly interacts with and localises to ATXN1 in human SCA1 neurons, where it ubiquitinates mutant ATXN1 in a chaperone-dependent manner, inducing degradation. These results demonstrate the therapeutic potential of CHIP for SCA1. The Notch intracellular domain (NICD) might also enhance the degradation of ATXN1 through MDM2-mediated ubiquitination (Kang et al., 2017). In addition, some ubiquitin-like proteins, such as A1Up and FAT10, potentially regulate the proteasomal degradation of mutant ATXN1 (Davidson et al., 2000; Hipp et al., 2005), but the specific mechanism still requires further exploration. One study (Guo et al., 2014) has shown that PQC systems, including PML (promyelocytic leukaemia protein) and RNF4 (a ubiquitin ligase), mediate the degradation of misfolded proteins including ATXN1. In this system, PML binds to the misfolded protein and conjugates it to poly-SUMO2/3 chains, and RNF4 ubiquitinates SUMOylated misfolded proteins, targeting them to the UPS. This system serves as another potential therapeutic target for neurodegenerative diseases, although its role in other SCAs still requires further verification.

ATXN3, a DUB containing the ubiquitin-binding UIM and a ubiquitin protease domain, suppresses polyQ neurotoxicity through degradation, although this function is partially impaired in pathogenic ATXN3 (Warrick et al., 2005). Ubiquitination has been reported to promote the DUB activity of ATXN3, which further contributes to Ub-dependent homoeostasis and neuroprotection in SCA3 (Todi et al., 2009). Parkin, an E3 ubiquitin ligase, promotes the ubiquitination and degradation of ATXN3, which is enhanced by Hsp70. Furthermore, parkin improves proteasome activity after impairment by misfolded polyQ proteins (Tsai et al., 2003), indicating that parkin might serve a protective role in SCA3. CHIP also promotes the degradation of ATXN3 by enhancing its ubiquitination, which would be increased by Hsp70 (Jana et al., 2005). Another ubiquitin-related protein is the ubiquitin chain assembly factor E4B. E4B also promotes the degradation of polyQ-expanded ATXN3 by enhancing ATXN3 polyubiquitination and conjugation to VCP (an AAA-type ATPase) to modulate a shift between ATXN3 and the proteasome. Consequently, the targeted expression of parkin, CHIP and E4B could be a novel therapeutic strategy for SCA3. To date, several sites on ATXN3 have been found to be ubiquitinated in mammalian cells (K8, K117, K190, K200, K206, and K29, with K117 and K200 being the primary sites) (**Figure 1B**), and there are no significant differences between the ubiquitination of normal and mutant ATXN3 except that ubiquitination on K8 is enhanced on mutant ATXN3, which might affect catalytic activity. In addition, the ubiquitination of K200 was not detected on mutant ATXN3 (Todi et al., 2010; Kristensen et al., 2017). The ubiquitination of ATXN3, primarily at site K117, activates the DUB function of ATXN3 through a conformational switch, which improves the editing of Ub chains on substrates. This activates Ub-dependent pathways, including the proteasomal degradation of misfolded proteins, and improves neuroprotective activity (Todi et al., 2010; Faggiano et al., 2015). Interestingly, Blount et al. (2014) found that ubiquitination is not absolutely necessary for ATXN3 proteasomal turnover, which is modulated by ubiquitin-binding Site 2 (UbS2) on the amino terminus. UbS2 might promote the interaction between ATXN3 and Rad23A/B, the proteasome-associated proteins, and this interaction might block ATXN3 turnover. Thus, occupation of the UbS2 binding site, preventing an interaction with Rad23A/B, would promote the proteasomal degradation of ATXN3 and further suppress neurotoxicity, suggesting a novel target for SCA3 therapy.

As a common process in polyQ neurodegenerative disorders, ubiquitination is commonly believed to be involved in DRPLA pathogenesis. In DRPLA brains, ATN1 forms an abnormal complex that is pathologically ubiquitinated to form neuronal cytoplasmic inclusions. In addition, this ubiquitination is discovered selectively in affected lesions (Yazawa et al., 1999). Through immunoblots of brain tissues from DRPLA patients, pathological ubiquitination is found to correlate with the size of an expanded glutamine repeat in ATN1 and the onset of symptoms. Thus, pathological ubiquitination of the ATN1 complex plays an important role in DRPLA pathogenesis. Interestingly, this abnormal complex is insoluble in water but soluble in SDS and reducing agents, unlike the characteristic solubility of abnormally ubiquitinated complexes in Alzheimer’s disease. This study reveals a unique pathological mechanism for DRPLA in which an insoluble complex is formed by spontaneous accumulation rather than by a qualitative change in water solubility (Yazawa, 1999).

Overall, an in-depth study of the aberrant ubiquitination in SCAs can facilitate a better understanding of the pathogenesis since UPS is the main pathway for protein clearance. Besides, targeting the various chaperons involved in ubiquitination to upregulate the ubiquitination of mutant proteins in SCAs may be a protective therapeutic strategy. Al-Ramahi et al. (2006) have demonstrated that CHIP overexpression lessens the toxicity induced by mutant ATXN1 in *Drosophila* models of SCA1 due to upregulation of ATXN1 ubiquitination. E4B has been verified to suppress the neurotoxicity of polyQ-expanded ATXN3 in *Drosophila* models of SCA3 by promoting the ubiquitination and degradation of mutant ATXN3 (Matsumoto et al., 2004). The targeted expression of specific ubiquitination interactors such as CHIP and E4B suggests potential gene therapy routes to treat SCAs.

## SUMOylation

SUMOylation which is a vital regulator of the proteostasis is the binding of SUMO (small ubiquitin-like modifier) proteins, including SUMO-1, SUMO-2, SUMO-3, and SUMO-4, to the lysine (K) residues of target proteins. SUMOylation can modulate proteostasis independently and usually enhances protein stability; however, the interaction between SUMOylation and ubiquitination can be cooperative or competitive and is a primary regulator of proteostasis (Liebelt and Vertegaal, 2016). Protein SUMOylation mediates the pathogenesis of multiple diseases, including neurodegenerative diseases (Yang et al., 2017); for example, the SUMOylation of HTT protein by SUMO-1 enhances its stability and contributes to the neurodegeneration observed in Huntington’s disease (Steffan et al., 2004). Here, we review the role of SUMOylation in the pathogenesis of SCAs.

In ATXN1, there are five lysine residues that are SUMOylated: K16, K194, K610, K697, and K746 (**Figure 1A**). The SUMOylation of ATXN1 is likely modulated by multiple factors, such as phosphorylation of Ser776, polyQ length, nuclear localisation and the self-association region (Riley et al., 2005). The SUMOylation of ATXN1 by SUMO-1 promotes ATXN1 aggregation, thus enhancing neurodegeneration in SCA1. Furthermore, incubation with hydrogen peroxide to stimulate oxidative stress accelerates SUMO conjugation to ATXN1 and enhances aggregation as a result, which could be modulated by the JNK pathway (Ryu et al., 2010).

In SCA3, ATXN3 can be SUMOylated at site K166 (Zhou et al., 2013) (**Figure 1B**) by SUMO-1, which would enhance mutant ATXN3 stability without affecting aggregate formation. In addition, ATXN3 SUMOylation by SUMO-1 on site K166 also increases apoptosis in SCA3; therefore, SUMOylation by SUMO-1 might stimulate SCA3 pathogenesis through both effects described above. In addition to K166, ATXN3 can also be SUMOylated at site K356 (**Figure 1B**) by SUMO-1 and SUMO-2. SUMOylation at K356 (Almeida et al., 2015) reduces the formation of ATXN3 aggregates and strengthens the association with the AAA+ ATPase p97, which can promote p97-mediated ER-associated protein degradation. Consequently, SUMOylation at K356 impedes ATXN3 aggregation through two pathways, further exerting a protective influence during SCA3 pathogenesis. We observed different effects when different ATXN3 sites were SUMOylated.

SCA7 is caused by the aberrant expansion of CAG repeat in the first exon of the ATXN7-encoding gene (David et al., 1997). ATXN7 is a subunit of the DUB module in the Spt-AdaGcn5-acetyltransferase (SAGA) complex, and this complex can regulate histone acetylation and ubiquitination to modulate gene expression (Koutelou et al., 2010). ATXN7 incorporates the expanded polyQ, a nuclear export signal (NES) and three NLS, in which the range of polyQ repeats is from 37 to 130 in SCA7 patients but from 7 to 35 in healthy individuals (David et al., 1998; Young et al., 2007) (**Figure 1C**). SUMOylation on K257 (**Figure 1C**) reduces the toxic aggregate production and apoptosis caused by expanded polyQ-ATXN7 without affecting ATXN7 subcellular localisation (Janer et al., 2010). Therefore, changing the SUMOylation status of SCA7 might reduce SCA7 pathogenesis.

SUMO-1 has been verified to co-localise with polyQ aggregates in both the DRPLA cellular model and brain tissue. Moreover, co-transfection of SUMO-1 with polyQ ATN1 dramatically enhances the number of intranuclear inclusions (NIs) and, consequently, neuronal apoptosis, whereas a non-conjugating mutant of SUMO-1 shows the opposite effect. These results reveal that SUMO-1 is involved in the DRPLA process and promotes polyQ aggregate formation and cell death (Terashima et al., 2002). Further exploration is needed to elucidate the specific mechanisms involved in the association between SUMOylation and DRPLA.

Serving as a critical regulator of proteostasis, SUMOylation can change the aggregation formation and degradation of mutant proteins in SCAs and whether the regulation is promotion or inhibition is related to the different amino acid residues. What’s more, SUMOylation has many more roles, such as regulating genome organisation, protein-DNA binding and DNA repair (Hickey et al., 2012). Whether these roles are involved in SCA pathogenesis still needs further investigation, and this complexity poses a barrier to transferring SUMOylation roles into therapeutic targets.

## Proteolytic Cleavage

Proteolytic cleavage is a type of PTM with important roles in neurodegenerative diseases. This process is catalysed by special proteases, such as the caspase and calpain families, and removes or separates the specific parts of protein sequences, turning long proteins into short fragments. Increasing evidence has suggested that fragments containing polyQ expansions are more likely to aggregate and cause toxicity than full-length polyQ-expanded proteins; this is known as the toxic fragment hypothesis (Matos et al., 2017). Here, we primarily discuss SCA3, SCA7, and DRPLA, in which stable proteolytic fragments are observed.

Schmidt et al. (1998) discovered that NIs in neuronal SCA3 patient cells are created by fragments of pathogenic proteins, implying that ATXN3 undergoes proteolytic cleavage prior to its transport into the nucleus. ATXN3, a protein with a molecular weight of approximately 42 kDa, is proteolytically modified by caspases and calpains (Berke et al., 2004; Weber et al., 2017). D241, D244, and/or D248 are the three most relevant caspase cleavage sites in ATXN3 (Evers et al., 2014), and the two major calpain cleavage sites occur at amino acids D208 and S256 (Weber et al., 2017) (**Figure 1B**). Short polyQ-expanded fragments of ATXN3 that aggregate are essential factors in the pathogenesis of SCA3 (Schaffar et al., 2004). The toxic polyQ-expanded fragments led to increased mitochondrial fission, decreased mitochondrial membrane potential and increased reactive oxygen species, causing cell death (Hsu et al., 2017). In addition, the structure of full-length ATXN3 can be distorted by polyQ-expanded fragments, causing them to interact with each other and form co-aggregates (Haacke et al., 2006). These observations led to the current consensus that ATXN3 fragments are more toxic than full-length ATXN3. Caspases involved in proteolytic cleavage are initially activated after signalling events. Then, the activated caspases cleave their target substrates, such as full-length ATXN3, after an aspartate residue (McIlwain et al., 2015). One study has shown that inducing caspase activity leads to increased aggregation and cleavage of a C-terminal 28 kDa ATXN3 fragment in cells expressing mutant ATXN3, which facilitates neurodegeneration (Liman et al., 2014). However, studies suggest that calpains have a greater influence on aggregate formation than caspases (Koch et al., 2011; Evers et al., 2014). When the endogenous calpain inhibitor calpastatin was knocked out to enhance calpain activity in SCA3 mice, increased aggregation and accelerated neurodegeneration were observed in the cerebellum (Hubener et al., 2013). Similarly, a study overexpressing the endogenous calpain inhibitor calpastatin to inhibit calpain activity in SCA3 mice showed decreased aggregation and nuclear toxicity (Simoes et al., 2012). Oral administration of the calpain inhibitor BDA-410 has the same outcome (Simoes et al., 2014). Thus, we conclude that calpains contribute significantly to the ATXN3 cleavage pathway.

ATXN7, a member of the STAGA complex, is involved in transcriptional regulation (Helmlinger et al., 2004). The specificity of neuronal cell death in SCAs may be correlated with caspase expression (Hermel et al., 2004). ATXN7 is responsible for the recruitment and activation of caspase-7 in the nucleus, which, in turn, cleaves ATXN7, contributing to the formation of Nis (Young et al., 2007). PolyQ-expanded proteins can activate caspase-7 *in vivo*, which cleaves ATXN7 at the D266 and D344 sites (**Figure 1C**) and generates an ATXN7 fragment that is more toxic than the full-length protein (Young et al., 2007; Guyenet et al., 2015). In addition to the toxic fragments arising from the polyQ-expanded protein, caspase-3 cleavage of amyloid precursor-like protein 2 (APLP2) may also contribute to SCA7 pathogenesis by producing intracellular C-terminal domains that might enhance aberrant transcriptional regulation (Takahashi-Fujigasaki et al., 2011). Thus, we conclude that caspase cleavage is a critical event in ATXN7 neurotoxicity and the pathogenesis of SCA7. In addition, the inhibition of caspase cleavage may provide a positive avenue for SCA7 treatment.

Multiple studies have shown that the caspase family is associated with DRPLA protein cleavage. The C-terminal polyQ-expanded fragment produced by proteolytic caspase-3 cleavage at D109 of ATN1 (**Figure 1E**) *in vitro* is responsible for the formation of aggregates and neurodegeneration (Miyashita et al., 1998; Ellerby et al., 1999). In DRPLA disease models and patient brain tissue, it has been demonstrated that ATN1 proteolytic processing modulates the intracellular localisation of fragments, which are also involved in DRPLA pathogenesis (Suzuki and Yazawa, 2011). Interestingly, the selective accumulation of the C-terminal ATN1 fragment is enhanced by the inhibition of caspases in COS-7 cells. This may arise from additional roles of caspases; for example, they are involved in the regulation of ATN1 (Suzuki et al., 2010). Further exploration is required to determine the specific mechanisms of caspases in DRPLA pathogenesis.

In brief, proteolytic cleavage could generate short polyQ-expanded fragments, causing more aggregation and toxicity in cells. Based on the existing studies, several therapeutic strategies could be considered. For one thing, altering the specific cleavage site of disease proteins could offer opportunities to reduce toxicity, thus affecting the neurodegenerative process. In SCA3, the identification of the ATXN3 cleavage sites D208 and D256 provided a potential technique by skipping exons containing the calpain recognition motif (Weber et al., 2017). For another, treatment with caspase and calpain inhibitors may also demonstrate their therapeutic potential. Calpastatin, a calpain inhibitor, decreases the nuclear uptake and aggregation of mutant ATXN3, delaying neurodegeneration in SCA3 mouse models (Simoes et al., 2012). However, this result does not coincide with the result of caspase inhibitors used in DRPLA disease models, suggesting that another signal pathway of caspases and calpains is involved.

## Transglutamination

Transglutamination is a PTM that results in an acyl shift between the γ-carboxamide group of a polypeptide-bound glutamine and the ε-amino group of a lysine residue in another protein, which is catalysed by transglutaminases (TGs) (Violante et al., 2001). TG families include three types of TGs (Lorand and Graham, 2003): papain-like TGs, bacterial toxin TGs and protein disulfide isomerase-like TGs. The primary function of mammalian TG is to modulate the formation of cross links between proteins for the stabilisation of biological structures (Aeschlimann and Thomazy, 2000). Because expanded polyQ tracts, which provide substrates for TGs, account for most common SCAs, TGs may play important roles in SCA pathogenesis.

Tissue-specific TGs have been demonstrated to exist widely in the rat and human CNS, such as in the brain, the cerebellum and in different classes of neurons (Maggio et al., 2001). TGs have been reported to facilitate mutant protein aggregation in SCAs, and the integration of polyQ AR with TG contributes to proteasome dysfunction (Kahlem et al., 1998; Mandrusiak et al., 2003); thus, TG can promote neurodegeneration in SCAs. ATXN1 has been demonstrated to be a substrate of TG type 2 (TG2), and SCA1 transgenic mice have showed elevated nuclear TG2 in the Purkinje cells of the cerebellum (D’Souza et al., 2006). In addition, TG2 mediates the recruitment of the calcium binding protein calbindin-D28k (CaB) to ATXN1 (Vig et al., 2007). The interaction between CaB and myoinositol monophosphatase (IMPase) is necessary for inositol-1, 4,5-trisphosphate (IP3)-mediated Ca^2+^ signalling, which is significantly involved in synaptic integration and plasticity in the peripheral nervous system (Schmidt et al., 2005). The recruitment of CaB to ATXN1 might decrease CaB levels and impede IP3 signalling, which contributes to SCA1 neurodegeneration.

To conclude, transglutamination is an important driver of SCA1 pathogenesis, possibly by modulating aggregate formation and CaB recruitment. Given the enzyme-substrate relationship between TG2 and ATXN1, further exploration on specific mechanism is needed as well as the effects of transglutamination in other SCAs.

## Acetylation

Acetylation is the process of covalently binding an acetyl group to a lysine residue on a protein and is regulated by two groups of enzymes: histone deacetylases (HDACs) and histone acetyltransferases (HATs) (Park et al., 2015). There have been 18 HDAC subtypes identified in humans, which include Class I HDACs (HDAC1, 2, 3 and 8), class IIa HDACs (HDAC4, 5, 7 and 9), class IIb HDACs (HDAC6 and 10), class III HDACs and class IV HDACs (Xu et al., 2007). Many studies show that acetylation plays diverse roles in neurodegenerative diseases.

Epigenetic dysregulation is an important characteristic of polyQ diseases (Cohen-Carmon and Meshorer, 2012). Nucleosome is the basic unit of chromatin, which incorporates 147 base pairs of DNA enwinding an octamer of core histones (H2A, H2B, H3, and H4) (Quina et al., 2006). The equilibrium between the acetylation and deacetylation of histones, which occurs at the N-terminal tails of histones [H3 is acetylated at K9, K14, K18, and K23; H4 is acetylated at K5, K8, K12, and K16 (Carrozza et al., 2003)], modulates gene transcription by changing the interaction between histones and DNA as well as nuclear proteins (**Figure 2B**) (Kornberg and Lorch, 1999). The mutant proteins containing expanded polyQ can suppress the histone acetylase activities of some acetylation-related interactors, such as CREB-binding protein (CBP), p300 and p300/CBP-associated factor (P/CAF), through direct interaction with their acetyltransferase domains, which further inhibits histone acetylation and leads to epigenetic dysregulation (Hughes et al., 2001; Steffan et al., 2001). Aberrant histone acetylation and epigenetic dysregulation have been verified in a variety of SCAs. Among them, ATXN1 is connected to leucine-rich acidic nuclear protein (LANP), an ATXN1-binding co-repressor, and can inhibit the activity of the histone acetyltransferase CBP (Cvetanovic et al., 2012). ATXN3 has been verified to inhibit the acetylase activities of CBP and p300 *in vitro* through protein–protein interactions (Li et al., 2002). ATXN7 is a constituent of the SAGA complex, which acetylates targeted histones. Furthermore, mutant ATXN7 disrupts structural integrity and interferes with the substrate targeting of the SAGA complex, further affecting histone acetylation (McCullough and Grant, 2010). Therefore, reversing histone hypoacetylation by treating patients with HDAC inhibitors might alleviate neurodegeneration symptoms.

In addition to the acetylation of histones, acetylation at the K257 residue of ATXN7 (**Figure 1C**) enhances the accumulation of the caspase-7 cleavage product by inhibiting protein turnover, and accumulation of this fragment promotes SCA7 pathogenesis (Mookerjee et al., 2009). HDAC3 increases K257 acetylation through a direct protein–protein interaction with ATXN3 instead of through deacetylase activity, which contributes to SCA7 disease progression (Duncan et al., 2013). Thus, inhibiting the acetylation of ATXN7 by inhibiting HDAC3 may slow SCA7 pathogenesis.

Taken together, abnormal histone acetylation might partially contribute to the pathogenesis of SCAs. The major mechanism is that polyQ expanded proteins suppress histone acetylase activities, leading to histone hypoacetylation. Therefore, altering the decreased expression of acetylated histones could be an effective therapeutic target in SCAs. In-depth studies on HDAC inhibitors have developed. There are several HDAC inhibitors, including sodium butyrate (SB), suberoylanilide hydroxamic acid (SAHA), and valproic acid (VPA), which have been shown to reverse histone hypoacetylation and improve motor performance in DRPLA, HD, and SCA3 mouse models (Ying et al., 2006; Mielcarek et al., 2011; Yi et al., 2013; Long et al., 2014). The safety and validity of VPA have been verified in SCA3 patients in a clinical trial, which showed that treatment with VPA improved patient locomotor function at a dose that patients could tolerate (Lei et al., 2016). However, the complex roles of acetylation in different proteins in SCA pathogenesis should be considered; thus, the targeted accuracy is truly essential.

## N-Glycosylation

*N*-Glycosylation, one of many glycosylation patterns, is another PTM related to SCA disease progression. The N-linked protein glycosylation process contains two phases, which occur in the ER and the Golgi apparatus (Marquardt and Denecke, 2003). The first phase is the transfer of a preassembled oligosaccharide to the asparagine of the motif Asn-X-Ser/Thr [the common glycosylation site, X, can be any amino acid but proline (Gavel and von Heijne, 1990)] of a protein in the ER, and this process is accomplished by oligosaccharyl transferase (OST), a multimeric enzyme complex. In the second phase, the removal of several monosaccharides and the addition of several units, including GlcNAc, sialic acid, galactose and fucose residues, occur in the ER and Golgi. A variety of enzymes are involved in the second phase, including mannosidases (I, II, III), galactosyltransferase, and sialyltransferase. N-linked protein glycosylation affects multiple cellular pathways, such as protein folding, quality control, degradation, and secretion (Marquardt and Denecke, 2003; Freeze and Aebi, 2005).

SCA13 is an infrequent autosomal dominant cerebellar ataxia caused by point mutations in *KCNC3*, which encodes the Kv3.3 voltage-gated potassium channel, and its symptoms include intellectual disability, seizure and ataxia. The Kv3.3 channel, a 757-amino-acid protein, contains six transmembrane segments and a pore re-entrant loop. Among these segments, the first four transmembrane segments (S1–S4) form the voltage sensor domain and the last two segments (S5–S6) compose the ion-selective pore (**Figure 1D**). In addition, this channel could facilitate the firing of vast action potentials per unit time in neurons (Waters et al., 2006). To date, several variants have been described, including the *R420H* variant, the *R423H* variant and the *F448L* variant (Figueroa et al., 2010). Two conserved *N*-glycosylation sites have been confirmed on neural Kv3.3 channels and are located in the S1–S2 extra-cytoplasmic loop: the NKT amino acid residues, located from 321 to 323; and the NIT amino acid residues, located from 337 to 339 (**Figure 1D**). The *N*-glycosylation status of Kv3.3 channels is thought to affect K^+^ currents at the neuronal surface, which may affect neurodegeneration (Cartwright et al., 2007). Gallego-Iradi et al. (2014) detected aberrant *N*-glycosylation of the Kv3.3 channel in the *R420H* variant (KCNC3^R420H^). They also found that KCNC3^R420H^ proteins were retained in the Golgi and exhibited limited trafficking to the plasma membrane because protein glycosylation is essential for transmission through the ER and Golgi. Aberrant Golgi and cellular morphology arise from this, which might be a cellular manifestation of SCA13 pathogenesis.

Overall, the aberrant *N*-glycosylation of KCNC3^R420H^ can result in the abnormal K^+^ currents of neurons and the reduced membrane expression of KCNC3^R420H^ along with the abnormal Golgi and cellular morphology. Studying *N*-glycosylation may help to explain the molecular mechanism behind SCA13 manifestations.

## Conclusion

We reviewed multiple PTMs that affect the pathogenesis of SCAs through various mechanisms (**Table 2**). By regulating protein homeostasis, including protein interactions and the subcellular distribution, aggregation, stability and clearance of proteins, PTMs play significant and complicated roles in the regulation of SCA disease development. For instance, PTMs reducing the stability and aggregation of mutant proteins or modulating gene transcription, can generate protective influences on pathogenesis, such as the phosphorylation of ATXN2 by Cdk5–p25 and the ubiquitination of ATXN1. On the other hand, some PTMs contribute to SCA pathogenesis by promoting the stability and aggregation of mutant proteins. Phosphorylation at S776 of polyQ-expanded ATXN1 and proteolytic cleavage at D266 and D344 of ATXN7 could serve as examples. Due to the diversity of roles in SCAs, the regulation of PTMs uncovers novel potential therapeutic targets. PTMs are regulated through multiple cellular signalling pathways involving various molecules (Ju et al., 2014), such as kinases, ubiquitin ligases, phosphatases, transcription and splicing factors; therefore, therapies targeting PTMs by interfering with these pathways could potentially relieve neurodegeneration in SCAs. Massive efforts have been put into achieving this aim by silencing the pathways that induce PTMs stimulating SCA pathogenesis or activating the pathways responsible for PTMs that suppress SCA pathogenesis. For instance, inhibiting the phosphorylation-relevant pathways by downregulating the interactors RAS, ERK1, ERK2, and MAPK is proven to suppress ATXN1 levels and neurotoxicity in *Drosophila* and mouse models (Park et al., 2013). VPA, an HDAC inhibitor that reverses histone hypoacetylation, has been reported as a well-known therapeutic candidate in SCA3 (Lei et al., 2016). Although substantial studies have verified the validity of these therapeutic strategies in cells and animal models, which yield hope for clinical application, the clinical safety and efficacy of these agents might still be a huge problem due to the inconsistency among *in vitro, in vivo*, and clinical trials. In addition, due to the possible different outcomes arising from the same PTM at different sites, how to ensure target accuracy is still a big challenge. Furthermore, inhibiting or activating the distinct molecules responsible for PTMs mainly aim to regulate different downstream pathways, playing a destructive or protective role in SCAs. Therefore, none of them can relieve the SCAs from genome levels radically, such as gene editing. This means that even if the feasibility of these therapies is validated in clinical trials, patients with SCAs will have to take lifelong medication. Nevertheless, the deeper understanding of PTMs mechanisms in SCAs could provide us more efficient therapeutic avenues to treat SCAs, and the therapies at PTM levels are promising as well as challenging and thus are worth pursuing further.

## Author Contributions

LW and KX made substantial contributions to conception and design of the review article and drafted the manuscript. ZC, BT, and HJ revised the manuscript. HJ agreed to be accountable for all aspects of the work. All authors read and approved the submitted version.

## Conflict of Interest Statement

The authors declare that the research was conducted in the absence of any commercial or financial relationships that could be construed as a potential conflict of interest.

## Footnotes

1 Neuromuscular disease center [Internet]. Saint Louis: Washington University in St. Louis; [updated 2018 June]. Hereditary ataxias: dominant; [updated 2018 May 25; cited 2018 Jul 1]; [about 5 screens]. Available from: https://neuromuscular.wustl.edu/ataxia/domatax.html
